# *Vagococcus fluvialis* isolation and sequencing from urine of healthy cattle

**DOI:** 10.1093/g3journal/jkaa034

**Published:** 2020-12-22

**Authors:** Silvia Giannattasio-Ferraz, Adriana Ene, Laura Maskeri, André Penido Oliveira, Edel F Barbosa-Stancioli, Catherine Putonti

**Affiliations:** 1 Departamento de Microbiologia, Instituto de Ciências Biológicas, Universidade Federal de Minas Gerais, Belo Horizonte, MG 31270-901, Brazil; 2 Bioinformatics Program, Loyola University Chicago, Chicago, IL 60660, USA; 3 Empresa de Pesquisa Agropecuária de Minas Gerais—EPAMIG, Uberaba, MG 1170-495, Brazil; 4 Department of Biology, Loyola University Chicago, Chicago, Chicago, IL 60660, USA; 5 Department of Microbiology and Immunology, Stritch School of Medicine, Loyola University Chicago, Maywood, IL 60153, USA

**Keywords:** *Vagococcus fluvialis*, cattle microbiota, urinary tract bacteria

## Abstract

While the gram-positive bacterium *Vagococcus fluvialis* has been isolated from the environment as well as fish, birds, and mammals, very little is known about the species. *V. fluvialis* is believed to be a probiotic in fishes. However, within mammals, it is more frequently isolated from infectious tissue, including on rare occasions human and livestock lesions. Prior to the study described here, *V. fluvialis* had never been found in healthy bovine animals. Here, we present the complete genomes of *V. fluvialis* UFMG-H6, UFMG-H6B, and UFMG-H7, novel strains isolated from urine samples from healthy bovine females. These are the first genomes of mammalian isolates and the first description of *V. fluvialis* from urine. The genomes did not encode for any known virulence genes, suggesting that they may be commensal members of the urine microbiota.

## Introduction

*Vagococcus fluvialis* is a gram-positive, catalase negative bacterium first isolated in 1974 from chicken feces and river water. While these first isolates were originally identified as members of the *Lactococcus* genus, 16S rRNA gene sequencing led to their classification as a new genus: *Vagococcus* (Collins *et al.* 1989). This species was detected in mammals in 1994, isolated from lesions in pigs, horses, cats, and cattle (Pot *et al.* 1994). *V. fluvialis* was first associated with human infections in 1997 (from blood culture, peritoneal fluid, and wound), however, these isolates differed from those collected from pigs (of an unknown clinical source) both phenotypically and by their SDS-PAGE profile (Teixeira *et al.* 1997). So far, only two other papers have described the circulation of the species in humans, one isolate from a root-filled tooth associated with periradicular lesions and the other an isolate from a rare infective endocarditis (Al-Ahmad *et al.* 2008; Jadhav and Pai 2019). With a distinct lipid pattern that has a high concentration of d-alanylcardiolipin (Fischer and Arneth-Seifert 1998), *V. fluvialis* was also described as a potential probiotic to fishes *in vitro* and *in vivo* with an immunomodulatory effect to the host and the protection against *Vibrio anguillarum*, an important fish pathogen (Román *et al.* 2012; Sorroza *et al.* 2012; Román *et al.* 2013).

There is a great lack of knowledge regarding this species. Relatively few genome assemblies are available for the genus, and prior to the study described here, only three draft *V. fluvialis* genomes were publicly available in GenBank. Here, we describe three new strains of *V. fluvialis* isolated from urine from healthy Gyr heifers: *V. fluvialis* UFMG-H6, UFMG-H6B, and UFMG-H7. Furthermore, our analysis found that only two of the previously published draft genomes are in fact *V. fluvialis*.

## Materials and methods

The sample collection was occurred in May 2019 from a Brazilian herd composed of pure-by-origin Gyr cattle at the Agricultural Research Company of Minas Gerais State (EPAMIG). The reproduction in this livestock is realized by fixed time artificial insemination, without the presence of bulls. All of the experiments were performed according to relevant guidelines and were previously approved by the Ethics Committee in Animal Experimentation of the Universidade Federal de Minas Gerais, Brazil (CEUA/UFMG—40/2019).

For sampling, the animal’s vulva was washed with soap and distilled water. Mid-stream urine was collected using a sterile 50 ml conical tube. The material was frozen and kept at –20°C until processing 48 hours later. Then 2 ml of aliquots were made, centrifuged and spread onto Lysogeny Broth (LB) agar plates. These plates were incubated overnight at 37°C and all of the individual colonies were picked and regrown in LB under the same conditions (overnight at 37°C). This process of plating and liquid growth was repeated at least 3 times to obtain pure colonies. Single colonies were then picked and grown in liquid LB media overnight at 37°C, under agitation.

DNA was extracted using the Qiagen DNeasy UltraClean microbial kit (Qiagen, Hilden, Germany) according to manufacturer’s instruction and quantified using a Qubit fluorometer. All of the isolates were identified by 16S rRNA gene sequencing using the 63F/1387R primer pair; sequencing was performed by Genewiz (New Brunswick, NJ) using each primer individually. The resulting sequences were queried against NCBI’s 16S ribosomal sequence database via BLAST. Three isolates from two different animals were identified as *V. fluvialis*. Next, the DNA was sent to the Microbial Genomic Sequencing Center (MiGS) (Pittsburg, PA) for whole genome sequencing. The DNA was fragmented using an Illumina tagmentation enzyme and the indices were attached using PCR. Sequencing was performed using the Illumina NextSeq 550 platform producing 1.75 (UFMG-H6), 1.39 (UFMG-H6B), and 1.78 (UFMG-H7) million pairs of 150 nucleotide sequences. Next, the raw reads were trimmed using Sickle v1.33 (https://github.com/najoshi/sickle) and assembled using SPAdes v3.13.0 with the “only-assembler” option for k values of 55, 77, 99, and 127 (Bankevich *et al.* 2012). After assembly, the contigs were inspected using Geneious Prime (Biomatters Ltd., Auckland, New Zealand), removing contigs less than 500 nucleotides in length. The genome coverage for each assembly was calculated using BBMap v38.47 (https://sourceforge.net/projects/bbmap/). Annotation was performed using the NCBI Prokaryotic Genome Annotation Pipeline (PGAP) v4.11 (Tatusova *et al.* 2016). Default parameters were used for each of the software tools, unless previously noted.

The draft genome assemblies were also examined for plasmid presence, resistance genes, secondary metabolites, prophage sequences, and the CRISPR/Cas system. To check if the strains have a plasmid, the Center for Genomic Epidemiology’s (CGE) tool PlasmidFinder v2.1 was used with the following parameters: 60% minimum coverage, 90% minimum identity threshold, and gram-positive database (Carattoli *et al.* 2014). To complement this analysis, raw reads were also uploaded to the webtool PLACNETw to confirm the presence/absence of plasmids (Vielva *et al.* 2017). Assemblies were screened for antibiotic resistance genes using the CGE tool ResFinder v4.0, using default settings (Bortolaia *et al.* 2020). Secondary metabolites were predicted using the antiSMASH tool, with the strict detection parameter specified (Blin *et al.* 2019). The tool IslandViewer 4 was used to predict genomic islands (Bertelli *et al.* 2017). Each assembly as well as each publicly available *V. fluvialis* assembly was uploaded to the tool PHASTER for prophage prediction (Arndt *et al.* 2016). PHASTER predicts incomplete, questionable, and intact prophage regions. Intact nucleotide sequences were queried against NCBI nr/nt viral database for taxonomic classification. The genomes were also screened to detect CRISPR/Cas arrays using the webtool CRISPRCasFinder (Couvin *et al.* 2018).

The 16S rRNA sequences for each of the bovine *Vagococcus* genomes were extracted from the assembled genomes and queried against NCBI’s rRNA_typestrains/16S_ribosomal_RNA database to retrieve 16S rRNA gene sequences from other *Vagococcus* species. The 16S rRNA sequence was also retrieved for *V. fluvialis* DSM 5731, as it is not represented in this database, and *E. faecium* DSM20477, to serve as an outgroup. The sequences were aligned using MAFFT v7.388 (Katoh and Standley 2013) a phylogenetic tree was derived using the FastTree v2.1.11 (Price *et al.* 2010) plug-in though Geneious Prime. The tree was visualized using iTOL v5 (Letunic and Bork 2016).

The average nucleotide identity (ANI) of *V. fluvialis* UFMG-H6, UFMG-H6B, and UFMG H7 was computed using the tool JSpeciesWS (Richter *et al.* 2016). Each draft assembly was compared to each other, the three publicly available draft genomes of *V. fluvialis* in the NCBI Assembly database, and all other *Vagococcus* strains available through JSpeciesWS. The three *V. fluvialis* draft genomes include: *V. fluvialis* NCDO 2497 (Accession no. GCA_003987575.1) and *V. fluvialis* DSM 5731 (Accession no. GCA_003337315.1), both isolated from chicken feces, and *V. fluvialis* bH819 (Accession no. GCA_900163795.1), which was isolated from cheese. ANI values were visualized using Python.

Based upon our *Vagococcus* ANI analysis, we found that *V. fluvialis* bH819 had an ANI of 75.6% to the two other previously published genomes for the species. This is well below the 95% ANI threshold expected for strains of the same species (Ramasamy *et al.* 2014). (This strain had been excluded from our 16S analysis as it did not have a complete 16S rRNA sequence.) The ANI analysis prompted our further investigation of this genome. Relative to other *Vagococcus* species in the JSpeciesWS database, *V. fluvialis* bH819 has an ANI value ranging between 67% and 72%. We also compared it to *V. carniphilus* (NZ_CP060720), which has a marginally larger ANI value of 76.37%. The bH819 genome is not a bad assembly; the genome is assembled in 22 contigs with 379x coverage. Thus, we do not believe that the low ANI values detected are due to assembly issues. Using the Similar Genome Finder through Patric v3.6.7 (Davis *et al.* 2020), the closest relative identified was *Vagococcus* sp. strain UBA11317 (Accession no. GCA_03535935.1). ANI was calculated comparing this genome to *V. fluvialis* bH8019 identifying an ANI value of 99.96, suggesting that *Vagococcus* sp. strain UBA11317 and *V. fluvialis* bH8019 may represent a new species of *Vagococcus*. For our subsequent analysis of the three new bovine strains presented here, we removed *V. fluvialis* bH819 from consideration.

*V. fluvialis* UFMG-H6, UFMG-H6B, and UFMG H7 genomes were compared to the two ANI-confirmed, publicly available *V. fluvialis* genomes using the tool Anvi’o v6.2 (Eren *et al.* 2015). The pangenome was determined using the anvi-pan-genome function, using the NCBI BLAST option, setting the default minbit threshold of 0.5 and an MCL inflation value of 10. Single copy number core genes were identified and aligned using Anvi’o. The core genes were used to reconstruct a phylogenetic tree using the FastTree v2.1.11 (Price *et al.* 2010) plug-in though Geneious Prime and visualized using iTOL v5 (Letunic and Bork 2016).

### Data availability

All sequencing data are available in the NCBI Assembly database (www.ncbi.nlm.nih.gov/assembly) and short read archive (SRA) (www.ncbi.nlm.nih.gov/sra). The assemblies for *V. fluvialis* UFMG-H6, UFMG-H6B, and UFMG-H7 can be accessed with the following accession numbers: GCA_012102095.1, GCA_012102505.1, and GCA_012102415.1, respectively. The raw reads can be accessed with the accession numbers SRR11455641, SRR11455850, and SRR11455640 for UFMG H-6, UFMG-H6B, and UFMG-H7, respectively.

## Results and discussion

The genome statistics for the three *Vagococcus fluvialis* strains are shown in Table 1. None of the strains were found to contain a plasmid or to encode for antibiotic resistance genes. antiSMASH identified a putative bacteriocin encoded by *V. fluvialis* UFMG-H7. The presence of this putative bacteriocin may be related to the previously described probiotic potential of this specie (Román *et al.* 2012, 2013; Sorroza *et al.* 2012), although this hypothesis needs to be explored further.

**Table 1:** Genome statistics for *V. fluvialis* bovine isolates

	UFMG-H6	UFMG-H6B	UFMG-H7
Length (bp)	2,679,177	2,858,425	2,993,433
# Contigs	41	45	43
Genome coverage (x)	182	38	165
N_50_ score (bp)	135,292	112,454	131,353
GC content (%)	33.08	44.68	32.93
# Coding genes	2,626	2,792	2,906
# tRNAs	50	49	52

PHASTER results indicate the presence of an intact prophage in UFMG-H7; this prophage is 50.3 kb long, encoding 58 known proteins including terminase, integrase, tail, capsid, and portal proteins. PHASTER indicates that this prophage most closely resembles the siphovirus Bacillus phage BCJA1 (Accession no. NC_006557), and thus is likely a member of the *Siphoviridae* family. So far, this is the first predicted prophage infectious of *V. fluvialis*. This prophage does not closely resemble any previously characterized phage or prophage. Using discontiguous blast, only modest similarity was detected (<15% query coverage and <70% sequence identity).

Only one of the bovine *V. fluvialis* strains encodes for the CRISPR/Cas system; *V. fluvialis* UFMG-H6B has two spacer arrays and Cas type I-E genes. Cas type I-E was first described in *E. coli* genomes where the deletion of *Cas1* gene implied in a sensitivity to DNA damage [see review (Makarova and Koonin 2015)]. This is the first description of Cas type I-E in a *Vagococcus* strain; the Cas type II system (type A and type C) has been detected in other species from the genera (Pourcel *et al.* 2020)*.* IslandViewer analyses did not show any genetic islands or integrative elements for any of our *V. fluvialis* strains.

**Figure 1:**
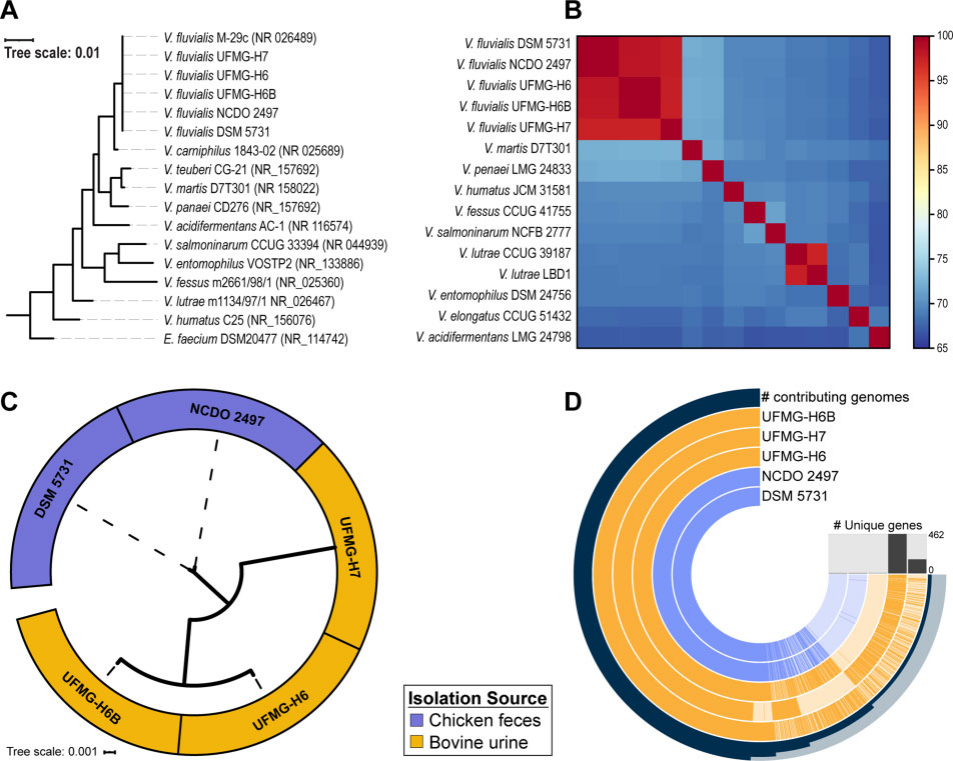
Comparison of five *V. fluvialis* draft genome sequences. (A) Phylogenetic tree of *Vagococcus* species based on the 16S rRNA sequence. (B) ANI comparison of *V. fluvialis* strains and other *Vagococcus* species. (C) Phylogenetic tree of *V. fluvialis* strains based on the core genome. (D) Pangenome of *V. fluvialis* strains. The three bovine urine isolates are shown in gold. Each gold and blue ring corresponds to a genome. Each ray corresponds with the presence (dark) or absence (light) of a given gene with the outer ring indicating the number of genomes that contain the particular gene. Genes were ordered according to this number. The histogram indicates the number of genes that are unique to each particular genome.

Our ANI analysis identified two publicly available *V. fluvialis* draft genomes in GenBank; an additional genome labeled as *V. fluvialis* is in fact not a member of this species (see *Materials and methods*), and was thus excluded from our analysis. 16S rRNA gene sequence and ANI analyses confirmed the species designation of *V. fluvialis* UFMG-6, UFMG-6B, and UFMG-7 (Figure 1, A and B, respectively). The core genome of these two confirmed *V. fluvialis* genomes and our three isolates was identified. It includes 3298 single copy number genes. The phylogenetic tree derived from this core genome shows that *V. fluvialis* UFMG-H7 is distinct from the other two bovine isolates, which clade together (Figure 1C). *V. fluvialis* UFMG-H6 and UFMG-H6B were isolated from the same animal, but they represent two different strains circulating within the urinary microbiota; their core genome has 92.73% sequence identity. Furthermore, the bovine urinary isolates clade separately from the two previously deposited genomes, both isolates from chicken feces.

Although built with only five genomes, *Vagococcus fluvialis* appears to have an open pangenome (Figure 1D). Further sequencing of isolates is needed to see if this holds true. 1143 genes are found within the accessory genome of the pangenome. The bovine urinary genomes encode for numerous genes not found within the two previously deposited genomes. Furthermore, UFMG-H7 includes 462 genes unique from the genomes of the two chicken fecal isolates and the two other bovine urine isolates. *V. fluvialis* has, however, been isolated from several other animals and environmental niches. Thus, sequencing isolates from these diverse sources is necessary to uncover the genic diversity of the species.

This is the first report showing *V. fluvialis* circulation in the urinary tract. The role of this bacteria in the urine microbiota is thus far unknown. However, it is important to highlight that, although previously isolated from lesions in cattle (Pot *et al.* 1994), none of the strains described here encode for genes associated with virulence factors, suggesting that these isolates do not have a pathogenic potential. Further *in vitro* evaluations of *V. fluvialis* UFMG-H6, UFMG-H6B, and UFMG-H7 would be necessary to understand if these strains have a probiotic potential, potentially related to the putative bacteriocin identified, and to look at their role in the urinary microbial community.
